# Supplementary material for: Auditory and sexual preferences for a father’s song can co-emerge in female Bengalese finches
Source: PLoS One. 2022 Mar 10;17(3):e0254302. doi: 10.1371/journal.pone.0254302 (PMC8912213; doi:10.1371/journal.pone.0254302)
Supplement: S1 File — A brief report of methods and results of a preliminary examination of the hormonal treatment. (DOCX) [file pone.0254302.s001.docx]

**S1 File. Effect of E2 implantation on CSD responses to song playbacks**

Previous studies in some songbird species have traditionally used estradiol (E2) implantation so that females will perform copulation solicitation displays (CSDs) to song playback alone [1]. To our knowledge, 3 studies reported that E2 implantation does increase CSD responses in Bengalese finch females [2–4], but the efficacy varied from one study to another. Therefore, we conducted a preliminary experiment to assess whether E2 implantation increases CSD expression in female Bengalese finches in our own experimental setting. For this purpose, we tested the behavior of 4 out of the 10 females used in the main experiment. Prior to the E2 implantation surgery and song preference tests described in the main text, these 4 birds went through 4 rounds of playback tests without hormone. Both pre- and post-surgery tests had the exact same schedule, song stimuli, method of stimulus presentation, environment and equipment. The results are summarized in Fig S1 and Table S1. In the pre-surgery tests, 3 birds showed no CSDs to any song stimuli. One bird (ID: B19Rd487) exhibited CSDs every time her father’s song was played but not when unfamiliar songs were played. In the post-surgery tests (the main experiment) however, all 4 birds performed CSDs to their father’s song and to one or two unfamiliar song(s). We calculated the response rate as the number of trials in which a bird performed CSDs divided by the total number of trials (including both the father’s and unfamiliar songs) and confirmed that the presence of the E2 tube increased the response rate (Fig S1). Therefore, we concluded that this hormone implantation method is functional in the current experimental paradigm and tested the remaining 6 birds after E2 implantation.

**
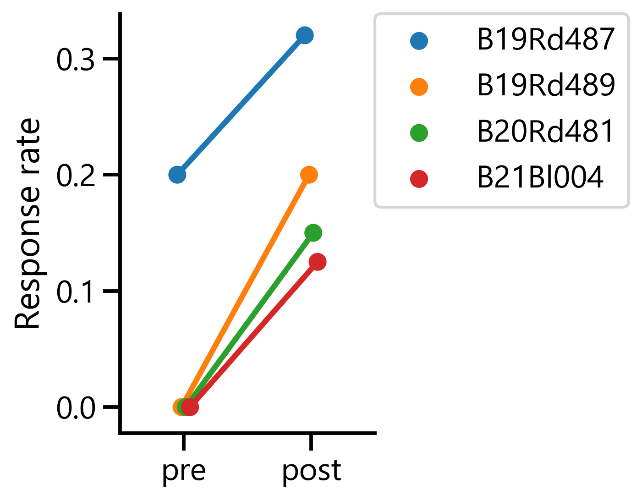
**

**Fig S1. A summary of CSD response rate before and after the E2 implantation**

The response rate was calculated by pooling all of the song stimuli presented in the tests.

| Subject ID | Stimulus | Pre-/post-surgery | Total tests | Total trials (A) | Responded trials (B) | Rate (B/A) |
| --- | --- | --- | --- | --- | --- | --- |
| B19Rd487 | Father | pre | 4 | 4 | 4 | 1.000 |
| B19Rd487 | unfamiliar | pre | 4 | 16 | 0 | 0.000 |
| B19Rd487 | father | post | 5 | 5 | 5 | 1.000 |
| B19Rd487 | unfamiliar | post | 5 | 20 | 3 | 0.150 |
| B19Rd489 | father | pre | 4 | 4 | 0 | 0.000 |
| B19Rd489 | unfamiliar | pre | 4 | 16 | 0 | 0.000 |
| B19Rd489 | father | post | 7 | 7 | 5 | 0.714 |
| B19Rd489 | unfamiliar | post | 7 | 28 | 2 | 0.071 |
| B20Rd481 | father | pre | 4 | 4 | 0 | 0.000 |
| B20Rd481 | unfamiliar | pre | 4 | 16 | 0 | 0.000 |
| B20Rd481 | father | post | 8 | 8 | 5 | 0.625 |
| B20Rd481 | unfamiliar | post | 8 | 32 | 1 | 0.031 |
| B21Bl004 | father | pre | 4 | 4 | 0 | 0.000 |
| B21Bl004 | unfamiliar | pre | 4 | 16 | 0 | 0.000 |
| B21Bl004 | father | post | 8 | 8 | 3 | 0.375 |
| B21Bl004 | unfamiliar | post | 8 | 32 | 2 | 0.063 |

**Table S1. CSD responses of 4 birds in pre- and post-surgery tests**

The responses of each bird to each song type (father’s and unfamiliar songs) based on the number of trials. The column ‘Total trials’ refers to the total number of trials (song playbacks) included in a series of tests. As we presented 4 different unfamiliar songs to each subject, the number of trials is 4 times larger than the number of tests.

**References**

1. Searcy WA. Measuring responses of female birds to male song. In: McGregor PK, editor. Playback and studies of animal communication. New York: Plenum Press; 1992. p. 175–89.

2. Okanoya K, Honda E, Nishikawa N. Time-series analyses of species specific songs in birds. Trans Tech Comm Psychol Physiol Acoust. 1998;28:1–6.

3. Clayton NS, Pröve E. Song discrimination in female zebra finches and bengalese finches. Anim Behav. 1989;38(2):352–4.

4. Dunning JL, Pant S, Bass A, Coburn Z, Prather JF. Mate choice in adult female Bengalese finches: females express consistent preferences for individual males and prefer female-directed song performances. PLoS One. 2014;9(2):e89438.
