# Supplementary material for: Auditory and sexual preferences for a father’s song can co-emerge in female Bengalese finches
Source: PLoS One. 2022 Mar 10;17(3):e0254302. doi: 10.1371/journal.pone.0254302 (PMC8912213; doi:10.1371/journal.pone.0254302)
Supplement: S2 File — A summary of the number of times each stimulus was presented to each subject. (DOCX) [file pone.0254302.s002.docx]

**S2 File. Additional information regarding song stimuli**

In the song playback tests, songs recorded from a total of 19 males were used. Each female was presented with 5 songs (1 father’s song + 4 unfamiliar songs) from this pool of 19 songs. Each father’s song was presented as an unfamiliar song for other subjects, but there were some variations in the total number of times a song was used. In addition, stimulus sets for different individuals sometimes shared unfamiliar songs. To precisely describe these variations and overlap, the song stimulus IDs are summarized as follows. In these tables, stimulus identities are represented by 19 alphabetical letters. The uppercase letters (A to G) are the songs of the subjects’ fathers. The lowercase letters (h to s) are the songs of other males that were unfamiliar to all females.

**Table S2-1. A list of stimulus sets (sorted by subject ID)**

Each row displays the stimulus set used for a subject.

| Subject ID | Father ID | unfam1 ID | unfam2 ID | unfam3 ID | unfam4 ID |
| --- | --- | --- | --- | --- | --- |
| B20Rd481 | A | B | i | n | P |
| B21Bl004 | B | C | j | o | Q |
| B19Rd487 | C | B | k | l | R |
| B19Rd489 | C | h | l | k | s |
| K27Bk016 | B | D | F | C | A |
| K29Rd003 | D | A | E | h | p |
| B25Rd008 | E | F | D | m | q |
| K31Rd013 | F | D | B | C | r |
| K31Rd011 | F | h | E | G | j |
| B26Rd014 | G | h | B | F | s |

**Table S2-2. A list of the number of times each stimulus was used (sorted by stimulus ID)**

The number of times each song was presented either as the father’s song, or an unfamiliar song are shown. Total counts are in the righthand column. These numbers correspond to the number of times each stimulus ID appears in Table S2-1 above.

| Stim ID | As father's song | As unfamiliar song | Total |
| --- | --- | --- | --- |
| A | 1 | 2 | 3 |
| B | 2 | 4 | 6 |
| C | 2 | 3 | 5 |
| D | 1 | 3 | 4 |
| E | 1 | 2 | 3 |
| F | 2 | 3 | 5 |
| G | 1 | 1 | 2 |
| h | 0 | 4 | 4 |
| i | 0 | 1 | 1 |
| j | 0 | 2 | 2 |
| k | 0 | 2 | 2 |
| l | 0 | 2 | 2 |
| m | 0 | 1 | 1 |
| n | 0 | 1 | 1 |
| o | 0 | 1 | 1 |
| p | 0 | 2 | 2 |
| q | 0 | 2 | 2 |
| r | 0 | 2 | 2 |
| s | 0 | 2 | 2 |
| Total | 10 | 40 | 50 |
