# Supplementary material for: Auditory and sexual preferences for a father’s song can co-emerge in female Bengalese finches
Source: PLoS One. 2022 Mar 10;17(3):e0254302. doi: 10.1371/journal.pone.0254302 (PMC8912213; doi:10.1371/journal.pone.0254302)
Supplement: S3 File — The total number of CSD occurrences in each test throughout the experiment is shown for each bird. (DOCX) [file pone.0254302.s003.docx]

**S3 File. Response frequency of each bird**


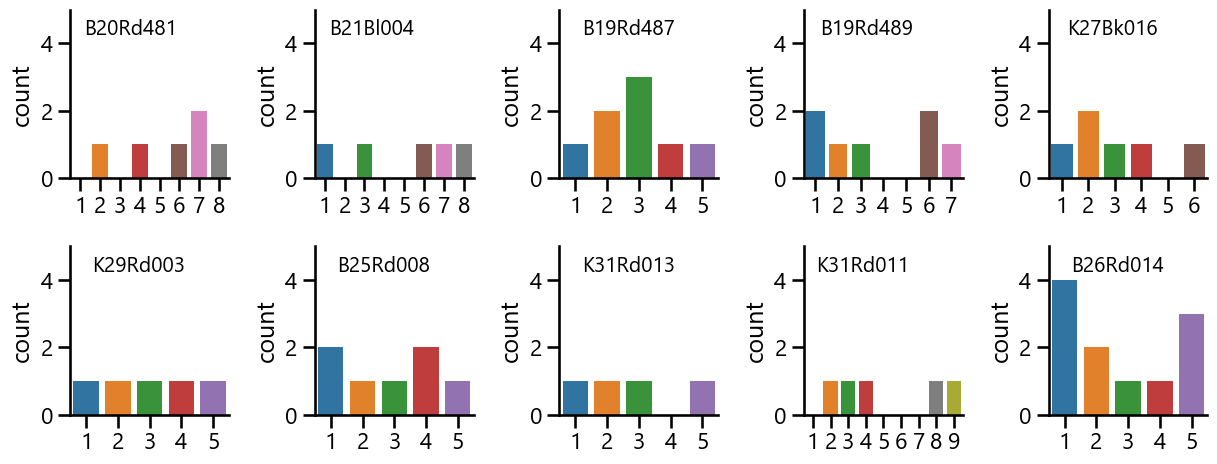


In the graphs above, the total number of CSD occurrences in a test is plotted against the test number (i.e., from day 1 to day 5-9, depending on the individual). Responses to all kinds of stimuli were pooled within a test. Each panel show data from one female. There was no systematic increase or decrease in the frequency of displays throughout the test series.
