# Supplementary material for: Auditory and sexual preferences for a father’s song can co-emerge in female Bengalese finches
Source: PLoS One. 2022 Mar 10;17(3):e0254302. doi: 10.1371/journal.pone.0254302 (PMC8912213; doi:10.1371/journal.pone.0254302)
Supplement: Attachment — Submitted filename: Fujii&Okanoya_PONE_Response to Reviewers.docx [file pone.0254302.s004.docx]

**General comments:**

We appreciate the reviewers’ and editor’s thoughtful and helpful comments on our manuscript. We acknowledge that our subject females may have developed under unusual social conditions due to a unique laboratory setting. As pointed out by the reviewers and the editor, the rearing regime should be clearly specified in the text, and the results must be interpreted more carefully. Thus, we revised the introduction, discussion, and related sentences in other sections to clarify the limitations of the current study as well as its contributions to the current body of research. Specific responses to each comment and the modifications made are described below. Original comments and questions from the reviewers/editor are italicized. All page and line numbers which specify the location of changes refer to the marked-up version of our revised manuscript unless otherwise mentioned.

**Academic editor:**

*> In particular, I agree with the reviewers that it is important to discuss the scope of interpretation based on the limited rearing regimes in the study. Reviewer 2's comment about the nature of the rearing conditions and external validity are important to discuss, as well as the potential for genetic (experience-independent) contributions to preferences. I also agree that details about the tutoring regime should be included in the title and abstract and that more of the Introduction and Discussion should be devoted to contextualizing your findings into the broader literature.*

Thank you for the important suggestions. According to the comment, we additionally discussed the limitations that may come from the unusual tutoring conditions (p. 19, lines 312-313; pp. 20-21, lines 334-347) and included rearing details in the title and abstract. We also revised the introduction and discussion sections to explain the purpose (p. 5, lines 62-67) and results of our study in relation to the previous findings reported in the broader literature (pp. 19-20, lines 324-328). We hope that our description now provides a sufficient amount of detail. Please also refer to our responses to similar comments raised by reviewer 2 (pp. 4-5 of this letter).

**Journal requirements:**

*> 1. Please ensure that your manuscript meets PLOS ONE's style requirements, including those for file naming.*

We ensured that the format and style meet PLOS ONE’s journal requirement.

*> 2. To comply with PLOS ONE submissions requirements, in your Methods section, please provide additional information on the animal research and ensure you have included details on (1) methods of sacrifice, (2) methods of anesthesia and/or analgesia, and (16) efforts to alleviate suffering.*

(1) No animals were sacrificed by the authors during the course of this research. (2) We used local anesthesia for E2 tube implantation. The type of anesthesia and surgical procedures are described in (p. 8, line 124). (16) While revising the methods section in response to reviewer 2’s comment, we added a description of how birds were handled during the experiment (p. 10, lines 165-169).

*> 3. In your Methods section, please include a comment about the state of the animals following this research. Were they euthanized or housed for use in further research? If any animals were sacrificed by the authors, please include the method of euthanasia and describe any efforts that were undertaken to reduce animal suffering.*

After this study, all birds continued to be housed in our laboratory colony for additional research (p. 6, lines 100-102), and no animals were sacrificed.

*> 4. Please include captions for your Supporting Information files at the end of your manuscript, and update any in-text citations to match accordingly.*

We added the list and captions for the Supporting information files at the end of our manuscript and checked the in-text citations.

**Reviewer 1:**

*> Line 26: As pointed out in the discussion, given that the females were reared by their genetic fathers, this study did not look at the role of early-life experience on the development of preference for father’s song.*

As you pointed out, the role of early experience remains unclear until preference is tested in cross-fostering experiments. We modified the sentence so that it focuses on future directions rather than suggesting an experience-dependent mechanism for the current results (p. 2, lines 28-31).

*> Lines 48-49: At least one of the studies cited, Anderson (2009), showed that female swamp sparrows exhibit more CSDs to songs from their local population. Thus, that study also links preference to sexual responses and does not only show “general selectivity to a familiar stimulus”. The main difference I see between the current study and that of Anderson is the identity of the familiar songs to which females show preferential sexual responses (songs from native population vs. songs of fathers).*

Thank you. It might be unfair that we did not refer to Anderson (2009) here, as it already demonstrated the correlation between operant responses and CSDs at least when female swamp sparrows were tested with local/foreign song dialects. We added this reference and another related study (Holveck & Riebel, 2007) and changed the description accordingly (p. 4, lines 50-56).

*> Lines 48-49: I do not understand what the authors mean by “sexual response in the context of mate choice”. Do they mean sexual responses in general, without considering preference? Or that their study is outside the context of mate choice? Though the birds in their study were not asked to choose a mate, the findings can have implications for mate choice, as pointed out by the authors in the discussion.*

We intended to convey that, strictly speaking, song preference can only be interpreted as mate choice if the ecological validity of the response is justified by other behavioral indices (such as CSDs). We agree that the previous description was ambiguous and modified the sentence for clarity (p. 4, lines 52-53).

*> Lines 71-74: It seems from reading these lines that at age 120 dph, the females were separated from any males. However, in Figure 1 and elsewhere in the methods it is mentioned that the females were separated from males just a few days before the experiment (about 279 dph). Can the authors please clarify what type of contact (either social or acoustic) did the females have with males between 120 dph and the few days before the experiment? In addition, some other details were not clear to me. Were there at any point, after 120 dph, males in the same room which the females could hear? Were the fathers in the same room?*

We agree that the social/acoustic environment of the females after separation from their family was not clear enough in the original manuscript. From 120 dph to the beginning of the experiment, females were kept in a single-sex cage, but conspecific males were also kept in other cages placed in the same room. Thus, during this period, the females could hear and see males although they could not physically interact with them. However, we made sure that their fathers were moved to another room so that the subjects had no acoustical and social access to their fathers between120 dph and the start of the experiment. We added this description in the methods section (p. 6, lines 86-90).

*> Line 112: “one subject’s father” – does that mean that the father of every female was used as an unfamiliar bird for other females?*

We meant that each subject’s father’s song was presented to other subjects as an unfamiliar song. We changed the description to provide clarity (p. 8, line 134). Please also see our comment to a similar question from reviewer 2 (pp. 8-9 in this letter).

*> Line 169: I do not see any reason why experience-independent preference for father song would not result in a correlation between preference and similarity to father’s song.*

As you pointed out, the description was not appropriate because the same prediction can be made regardless of the mechanisms underlying preference for the father’s song in females (either genetic or experience-dependent). We changed the sentence to remove the mention of experience-dependent developmental mechanisms here (p. 12, lines 208-209). We also changed the term in another sentence where we irrelevantly used ‘experience’ (p. 8, line 136).

*> Line 237: Could the lack of statistical significance be due to unfamiliar songs being not that similar to father’s song? (maximum similarity seems just a bit above 60%).*

Yes, that possibility cannot be excluded. We added this point in the discussion section (p. 21, lines 351-352). Thank you for your suggestion.

*> Line 282: The authors may want to compare their results to those of Riebel and Smallegange (2003; Journal of Comparative Psychology, 117(1), 61–66), who found that female zebra finches who have a preference for father’s song do not prefer sibling’s song, despite being similar.*

While answering your question above (to line 237 in the original manuscript), we also cited Riebel & Smallegange (2003) to indicate the possibility of using the brother’s song in future studies (p. 21, lines 357-362).

*> Figure3: The data used to generate these figures have not been disclosed.*

We uploaded all the data used to generate figures to the figshare depository. For Fig. 3, we used the dataset named ‘Fujii&Okanoya_PONE_Dataset2 (song similarity).xlsx’. It is available from the URL below. <https://figshare.com/s/dee039e4ac403fbd2a7e> (doi: 10.6084/m9.figshare.14677572)

**Reviewer 2:**

*> This is an interesting but also difficult to interpret finding – given that such a strong (almost exclusive) sexual preference for fathers’ song is highly unusual. As discussed below, this finding should perhaps be discussed more in the context of the socially unusual rearing situation. It seems also important that the authors acknowledge and discuss the lack of a control group of females raised in a different social setting to make sure that this is indeed a feature of Bengalese Finch preference development rather than a specific outcome of a specific laboratory rearing condition.*

Thank you very much for the important suggestions. We acknowledge the limitation of our study that the preference for father’s song found here cannot be generalized without testing birds reared in more natural settings with multiple social tutors and/or shorter periods of interaction with the tutor(s). Thus, we revised the discussion section (mainly in pp. 20-21, lines 334-347) and the related sentences in other sections so that the results can be interpreted more carefully.

Please let us clarify our standpoint to ensure correct communication hereafter: Our primary purpose was to test whether the preference to father’s song, **if any**, is sexually motivated. We do not intend to show that such a strong preference for father’s song is a general phenomenon that is expected to occur in this species under any rearing conditions. It has already been shown with other behavioral indices that female adult Bengalese finches (and zebra finches) respond more to their (foster) father’s song compared to unfamiliar conspecific songs (Kato et al., 2010; Fujii et al., 2021). As it was previously unclear whether this preference can be interpreted as a sexual preference for a male who sings the song, we designed the current study. In the previous manuscript, we stated that ‘Bengalese finch females prefer their **father’s song**’ because we thought we could not disentangle the genetic and experience-dependent effect on the development of that preference. We rather believe that the early-life social/auditory experience with adult male(s) should be critical to the development of song preference, and thus mating preference would be affected by multiple songs that females are exposed to early in life, if multiple male tutors are available. Through revising the manuscript, including the title and abstract, we attempted to disambiguate these points.

*> Could you provide more background information on:*

*1) the availability of song models in normal upbringing – both in the ancestral white-backed munia and in group housed domesticated Bengalese Finches? This would greatly help readers to interpret the findings and form an opinion on the question of whether*

*- the strong preference for the father’s song is an artefact of the rearing conditions or whether this would this happen with other rearing conditions as well.*

*- what is known about the sensitive phase for song memorisation in males and females in this species? And when would they normally leave the family group?*

*- earlier work on this species is poorly cited and discussed – there is substantial work on cross fostering and the development of mating preferences in this species that is not cited here but might shed light on when preferences develop?*

We appreciate the reviewer’s careful reading and helpful suggestions. In the previous manuscript, we did not provide enough information on the ecology of the Bengalese finch and white-rumped munias, including song learning processes. As you suggested, we cited additional references about the song learning process in male juvenile Bengalese finches (Clayton, 1987; Soma et al., 2009; Takahasi et al., 2010; Tachibana et al., 2017) and preference development in female zebra finches (Clayton, 1988; Holveck & Riebel, 2014) in the discussion section (pp. 20-21, lines 334-347). To our knowledge, however, there are very few studies that investigated the development of mating preference in female Bengalese finches. Although researchers have conducted many cross-fostering experiments in songbirds since the late 20^th^ century, Bengalese finches were usually used as foster parents of other species such as zebra finches. Also, it is difficult to specify the exact age of independence and availability of song tutors in wild white-rumped munias. Although knowledge of their ecology is limited, we provided a little more information about the social environment of this wild species (pp. 19-20, lines 324-328). We hope that this elaboration with additional references on laboratory studies in male Bengalese finches and other species of females aids in interpreting the findings of the current study.

*> 2) Should there have been a control group where females are exposed to more social song tutors in the form of a more social housing or the possibility to socialise/disperse at an age where this would normally happen?*

We do agree that rearing female birds in a more natural setting is an essential step in order to elucidate how song preference develops through experience. However, we believe this is outside the scope of our experiment, as the current study only examined whether the preference for father’s song can be interpreted as mating preference. Thus, we revised the manuscript so that the discussion section focuses more on the study limitations and the importance of future studies to approach these remaining issues (pp. 20-21, lines 334-347). In addition, the housing conditions in the current study (keeping family members together until the juveniles reach 120 dph) were set to match the conditions used in the previous studies which reported a preference for the father’s song in female Bengalese finches using operant conditioning or phonotaxis (Kato et al., 2010; Fujii et al., 2021). We also explained this in the introduction and method sections (p. 4, lines 66-67 & p. 5, 81-82).

*3) Looking at the results and in more detail on the data file suggests that overall responsiveness was low overall – but interpreting the data is difficult as the copulation solicitation displays were coded binary, i.e., we do not see the number of CSD given, only whether within a trial a female did or didn’t show any CSDs. Why was this coding chosen and is the father still the most preferred if the absolute number of CSDs for particular songs are analysed instead of the number of trials with CSDs? Do the number of CSDs observed here compare in their magnitude to what other studies in this species reported during song playback tests?*

Thank you for your question and comment. The reason we originally only used binary coding was that it was difficult to compare the number of CSDs due to individual variations in the duration of each CSD bout (please refer to the movies uploaded to the figshare repository for examples). Thus, in addition to the response frequency, we decided to analyze the total duration of CSDs during song presentation and compared the duration between the father’s song and unfamiliar songs for 7 out of 10 birds that responded to at least one unfamiliar song (method: p. 12, lines 194-196 & 201-206, result: p. 15, lines 260-265 and Fig. 2 (b)). Most birds (6 out of 7) showed longer displays to the father’s song than to unfamiliar songs although this tendency was not statistically significant. We believe this additional analysis along with response frequency data provides greater insight into song preference than frequency data could alone.

Please also note that although the overall response frequency was low, the response rate to the father’s song was relatively high: the response rate to the father’s song was the highest in 10 birds, and 4 out of them exhibited CSD(s) every time they were exposed to the father’s song. This suggests a high selectivity of response even when analyzed with frequency alone. As we explain in p. 10 in this letter, no trials were excluded from any statistical analysis or figure illustrations. The response rate reported in the text and illustrated in Fig. 2 (a) was calculated from all trials we conducted.

For the last part of your question, we think that the response frequency or magnitude of CSDs in our subjects was comparable to the results of similar studies. For example, Clayton & Pröve (1989) recorded CSDs during 12 minutes of song presentation in E2-implanted female Bengalese finches. They reported that the population mean of the total CSD frequency was about 20 times for conspecific song presentation, while the mean frequency was much less (~ 5 times) for heterospecific songs. In another study (Dunning et al., 2014), the authors tested E2-implanted females (12 birds) with 8 different unfamiliar conspecific songs for each individual. Although the exact number of songs was not described, they reported that all females responded to at least one song, but many songs evoked no CSDs at all. Although a direct comparison must be done with caution due to differences in conditions and experimental environment, it seems that the frequency of CSDs substantially depends on the type of song stimulus and can be very low particularly when presented with unfamiliar songs. We briefly referred to these previous studies in the discussion to help provide context for our results (p. 21, lines 352-354).

*4) A balanced discussion of the advantages of lab tutoring studies (high stimulus control, excellent approach for finding out about learning mechanisms and sensitive phases) versus their disadvantage (less well suited to find out how learning takes place in a socially and spatially more complex environment) should put the results in perspective. Other species might be informative here too: In zebra finches, another well studied estrildid finch, preference development (song learning and visual imprinting) seems to depend highly on social learning -here sufficient lab experiments have been conducted to suggest that there is a sensitive phase for acoustic and visual preference learning/imprinting but that learning can take place with the father as model if there is nobody else but is equally strong if other models are offered during that period.*

We acknowledge that discussing the advantages and disadvantages of laboratory experiments vs. studies in a more natural and complex social environment is very important, and was lacking in our original manuscript. We cited other references, including studies in zebra finches as you suggested, and other studies that investigated Bengalese finch song learning with multiple male tutors (pp. 20-21, lines 334-347).

*5) In view of the above I recommend adjusting the current title “Female Bengalese finches recognize their father’s song as sexually attractive” as for one it is semantically ambiguous: ‘they recognise” could mean that “they recognise it as sexually attractive” (leaving it unclear whether this is attractive to them or to other receivers) or that it is sexually attractive to them. Second, given the restricted social environment with the father as only adult song model up until 120 days the title should contain information indicating the context of a specific experimental rearing situation (at least hinting at the possibility that it is unclear whether the preference for the father’s song observed in this experiment could be an artefact arising from channelled learning mechanisms not receiving the input needed for normal development).*

We changed our title to avoid any ambiguity and to include more information on the rearing conditions of our subjects.

*More specific comments by line number:*

*> 30 ‘they can perceive..’ perhaps rephrase as it reads like a special talent but females are the intended audience of the species-specific mating signals!?*

*> 31 ‘may change’?*

It might be incorrect or misleading to say that female birds ‘perceive’ song variations. We revised this sentence to ‘they are sensitive to inter- and intra-species variations and may change their behavior…’ so that the message is more correctly conveyed to the readers (p. 3, lines 34-35).

*> 110 5 different songs: is this 5 different songs for each of the 10 females (i.e., 50 stimulus songs?) or did some females get the same test songs?*

All subjects were tested with a unique stimulus set, but there was some overlap between the songs used for each female. (Please see our response to your comment to line 112 for details.)

*> 112 ff Was each ‘father’ song used equally often used as unfamiliar control song? Please specify exactly how many test songs there were in total, how many of them were used with how many females and whether all fathers’ song were used as ‘unfamiliar songs’ as well (if this is yielding lengthy text, perhaps a (supplementary) table could summarise this?*

We acknowledge that more detailed information about these song stimuli is necessary and helpful for readers. As you suggested, we listed all the song stimuli used for each female in a table as a part of supporting information S2 File and updated the text (p. 8, lines 134-137). The total number of songs used as test stimuli is identical to the number of males used to record the song stimuli (19 birds), which is also shown in the Methods subsection ‘Animals’ (p. 6, lines 91-92).

*> 75ff can you give more information on the males – how were they reared? Were any of them siblings/raised by the same tutor?*

We added the rearing and family conditions of these males (as far as we know, since some birds were purchased from breeders). None of the birds were siblings, or tutored by the same male, which means all males used in these experiments had distinct songs and did not share song features (p. 6, lines 92-97).

*> 112 one father or each father?*

We meant ‘each father.’ We clarified it in the sentence (p. 8, line 134). Please also see the Supporting Information S2 File and our comments to your questions to lines 110 and 112 above.

*> 116 microphone was connected to..?*

*> 117 is this the soundcard or the recorder?*

The signals recorded from the microphone were processed through an amplifier and soundcard, then stored onto a PC. We changed the description to make it clear (p. 9, lines 141-143).

*> 119 when were songs recorded? Age? Before (days) or after breeding event?*

We added a bit more information about the timing of song recording in the Methods section (pp. 8-9, lines 138-141). At the time we performed song recordings, all the males (singers) were adult birds (> 180 dph). Because we could not tell the exact age of the males that were purchased from a breeder, we can only provide this lower limit. Also, songs of the subjects’ fathers were recorded before they were used for breeding. It is well known that the song features of Bengalese finches do not change depending on season, age or breeding experience, once the song is crystallized at about 120 dph (Okanoya & Yamaguchi, *J Neurobiol,* 1997; Tachibana, Koumura & Okanoya, *J Comp Physiol A*, 2015).

*> 121 please provide also sample size and range*

We added the sample size (as we used 6 renditions for each song, the total number of renditions were 6 * 19 = 114) and the rage of the stimulus duration (p. 9, lines 146-148). We also included the same information on the total duration of the 6 renditions for each singer.

*> 122 please specify – if you normalise the song by the standard deviation of the waveform (which one? The songs own waveform?) then each song will have another amplitude?*

The procedure was used to normalize the amplitude within one song (among the 6 renditions) and also between different songs. We added this description (p. 9, lines 150-151).

*> 128 please give the settings of the sound level meter*

We added the time weighting settings (‘fast’) at the end of the sentence (p. 9, line 156-157). The frequency weighting was set to type A, which is also specified in this sentence (p. 9, line 154).

*> 137 does this means that all other females were caught before another was tested? How often were females caught and moved in total during the test period? And with how many days in between catching/moving events?*

Yes, we handled the other females when moving them to the other soundproof chamber before testing a subject female. When moving them back to the cage after testing, we allowed the birds to fly back into the test cage without handling them. As we tested 1 bird or 2 birds in the morning of a day and each bird was tested once in 2 days, each bird was handled once or twice per day during the test period (ranged from 10 to 18 days). We added this information in the manuscript (p. 10, lines 165-169).

*> 143/44 please give the number of trials excluded (total, average per female/s.d.) but what was the rationale to not include all tests in the analyses? A female might not show any CSD’s because she was not responsive but it might also be an indication that she found none of the songs attractive enough?*

We did not exclude any trials from the analysis or the figure illustration (as written in the original manuscript p. 10, lines 163-164). We realized that it might appear as if we excluded the ‘invalid’ trials if we use the terms ‘valid/invalid.’ To avoid confusion, we changed the sentences that included these terms (pp. 10-11, lines 175-178).

*> 162 Even if there was individual variation: could you give the criteria you used to label females’ behaviour as a CSD, if not replication or comparison with other data becomes difficult.*

We identified the behavior as a CSD if a female showed all the following movement features: tilted head and chest down, tail raised and quivering. We revised this part of the manuscript to so that the criteria are clearer and easier to understand (p. 11, lines 191-193).

*> 191 Females were tested 10 times in a row so this variable has a time and order aspect, so should this not be a covariate? If it is analysed as a random variable you lose the information on the order (progress of time of testing) but it is likely that females’ responsiveness increased (if they got better acclimatized or if song further stimulated the reproductive system) or that it decreased if they started to dishabituate*

As you pointed out, the progress of time during the experiment might increase the response due to acclimation to the environment or decrease the response due to habituation to the stimuli. However, it is also possible that these effects interact or that there are individual differences in how these effects work. The data actually show that the number of tests required to reach the criterion of 5 responses varied between individuals. Thus, we found it difficult to assume a simply positive or negative effect of time on the response frequency. For those reasons, we decided to include this variable as a random effect rather than a fixed effect.

*> 219 was there an interaction between song type and presentation order?*

Although we did not include the interaction between song type (unfam/father = 0/1) and presentation order (1~5) in the original GLMM analysis, it may be reasonable to assume such an interaction. Here, we plotted the response frequency against either the song type or presentation order with logistic regression curves. Two figures show exactly the same data in 2 different ways. As you can see in the figures, it can be speculated that the effect of song type is more significant in later trials than in earlier trials, or in other words, the effect of presentation order is more significant in trials of unfamiliar songs than the father’s song.

Thus, we ran another GLMM analysis, in which all parameters were the same as those written in the original manuscript except that the interaction term (song type x presentation order) was additionally introduced. The estimated parameters are shown in the table below. However, the model estimation resulted in a singular fit, probably due to our limited samples. Because the primary purpose of this model analysis is to statistically describe the effect of song type, and the effect related to presentation order is an inevitable artifact due to experimental design, we think that a simpler, non-overfitted model (the original one) better suits this purpose rather than a more complex model that may lack generality.

| Independent variables | Estimates |  | *z*-value | *p*-value |
| --- | --- | --- | --- | --- |
| Fixed effects | coefficient | *s.e.* |  |  |
| intercept | -0.991 | 0.682 |  |  |
| **song type (unfam/father = 0/1)** | **2.287** | **0.928** | **2.464** | **0.014** |
| **presentation order (1-5)** | **-0.770** | **0.245** | **-3.140** | **0.002** |
| **interaction (song type × order)** | **0.857** | **0.331** | **2.585** | **0.010** |
| Random effects | variance | *s.d.* |  |  |
| subject ID (10 levels) | 1.064 | 1.031 |  |  |
| stimulus ID (19 levels) | 0.000 | 0.000 |  |  |
| test number (9 levels) | 0.208 | 0.456 |  |  |

*> Table 1: presentation order is here 1-5, but there were 10 test days, could you clarify this?*

*And there is also a random variable test number with 9 levels. Have you now not coded the same variable in two ways? I think the model should only contain the test day or test order – with so few birds and so many factors this model seems potentially overfitted?*

The presentation order here means the order of 5 stimuli **presented** **within a single test,** not the order of the test date. As described in the Methods section, one test was composed of 5 presentations of different song stimuli. Thus, we are sure that the model does not contain the same variable coded in two ways. In constructing the statistical model, we plotted the birds’ response against the variables that possibly affect the response frequency and found that a song stimulus tended to elicit more responses when presented earlier **within a test**. Thus, we thought that this parameter is not negligible and should be included in the model not to overestimate the effect of stimulus similarity on CSD responses.

*> 259/60 in view of the rearing conditions (father as only model until day 120) being so different from a normal upbringing, I suggest to phrase this conclusion more carefully*

We acknowledge that this is an important point. We stated here that the results need careful interpretation and discussed the limitations of the current study in later paragraphs (p. 19, lines 312-313).

*> 267 how would selection take place on the population level – the fitness advantage of individual mating preferences is on the individual level?*

We changed the term ‘species’ to ‘individual’ as it was confusing and inappropriate (p. 19, lines 320-322). As you point out, we did not mean that the selection works at the species level but wanted to convey that an individual who disfavors her father’s song may be able to avoid inbreeding.

*> 287 for the benefit of the reader: please specify what the previous findings in ref 14 & 15 were and how they support your argument.*

We inserted a brief description about these references as we agree that the previous manuscript was not informative enough for the readers (p. 22, lines 364-365).
