# Supplementary material for: Auditory and sexual preferences for a father’s song can co-emerge in female Bengalese finches
Source: PLoS One. 2022 Mar 10;17(3):e0254302. doi: 10.1371/journal.pone.0254302 (PMC8912213; doi:10.1371/journal.pone.0254302)
Supplement: Attachment — Submitted filename: Response to Reviewers(Fujii&Okanoya_PONE)round2.docx [file pone.0254302.s005.docx]

**General comments:**

Thank you very much again for your careful reading and suggestions. We now revised the manuscript to respond to the major and minor points raised by the reviewers. Because we needed to adjust the number of words in the abstract, we also modified some sentences that were not directly related to the reviewers’ comments, but these changes are also highlighted in the marked-up version. Specific responses and details about our modifications are described below. Line numbers refer to *the marked-up version* of our revised manuscript, unless otherwise noted.

**Journal requirements:**

*Please review your reference list to ensure that it is complete and correct. If you have cited papers that have been retracted, please include the rationale for doing so in the manuscript text, or remove these references and replace them with relevant current references. Any changes to the reference list should be mentioned in the rebuttal letter that accompanies your revised manuscript. If you need to cite a retracted article, indicate the article’s retracted status in the References list and also include a citation and full reference for the retraction notice.*

> We added a URL for one reference [31], which was lacking in the previous version. As we cited one additional reference [34] and changed the order of citations, the reference numbers from 16 to 53 differ from the previous manuscript. Other than that, the references remain the same.

**Reviewer #1:**

*The authors have addressed my comments satisfactorily. The only remaining suggestion that I have is that they should summarize their findings about the correlation between similarity and preference in the Abstract. Other than that, I wish good luck to the authors.*

> We appreciate your comments in the previous and current round of the review. In the latest version, we revised the abstract so that it also refers to the results regarding the correlation between stimulus similarity and preference (p. 2, lines 29-30).

**Reviewer #2:**

*The authors have written a detailed reply taking care to respond to all issues. For many this is satisfactory but a few major and minor points remain to clarify.*

> Thank you again for your very helpful comments and suggestions. We acknowledge the importance of precise communication, especially regarding the research purpose and the conclusions inferred by the results. We also understand the need to carefully explain the specific rearing conditions in the current study. Please see the following responses to each comment.

*1) As the study misses a control group with different song experiences/crossfostering the abstract and title should perhaps better be adjusted to be unambiguous regarding the specific experimental (narrow) context? A (hypothetical, not ideally phrased yet) title like “Female BF show sexual displays towards father song if this was only song model they experienced when juvenile” would be clear in this respect (not these exact wordings but as an indication how the title could indeed summarise the study). Such an adjusted title would alert readers from the beginning that this experiment might not trace normal development - this is not per se a critique of highly controlled experimental setups, at the contrary they can be very informative regarding learning mechanisms etc, and thus provide insights in the mechanisms of social transmission/learning. However, they provide only limited insights regarding transmission direction/ model choice if the experiment offers no choice with respect to song models. As is, the title suggests insights into a general feature of BF song preference development but with the lack of alternative song models during development you have tested a highly specific case only – readers should be informed about this in the title and abstract.*

> We appreciate your suggestion for the title. While revising the manuscript, however, we felt that your suggested title might unnecessarily direct the readers towards a very limited interpretation of the manuscript. As we clearly described the research purpose and the limitations of the study in the revision, we felt a slightly broader title was warranted. Please let us clarify our points here again: It has already been shown in previous studies that female Bengalese finches express selective phonotaxis, vocalizations, or operant behaviors to their father’s song, when they were reared in a single family and exposed to the father’s song almost exclusively. Though this differs from a wild environment, it is a standard social condition for Bengalese finches, as they are a domesticated species. However, song preference measured by such general behavioral indices (such as phonotaxis mentioned above) does not necessarily mean that females are **sexually** attracted to the father’s song. As mentioned in the introduction, there are indeed some studies that showed the correlation between CSDs and other responses to male songs, but it is unclear if this is also the case for the father’s song specifically. We believe that our study provides important insights for interpreting the previous results and designing future research, as laboratory studies of song preference usually use subjects reared under similar conditions to our study. Thus, the preferences of such birds can be substantially influenced by developmental song exposure. Of course, it is unlikely that a strong preference for the father’s song would generally occur in birds reared in a more complex social environment, though such a situation is rather unusual for a domesticated species such as the Bengalese finch. We acknowledge that this is out of the current study’s scope and thus discussed the limitations in the abstract and the discussion section. In summary, in this paper, we do not argue that female BFs generally develop a strong preference for the father’s song, but would like test if such a preference can be interpreted as a sexual preference. Therefore, we would like to use the title (full & short) as follows:

- Full: Female Bengalese finches show selective sexual displays to their father’s song
- Short: Selective sexual displays to the father’s song in female Bengalese finches

*2) The justification given to code CSDs as a binary variable is that its duration varied and separate vs. long CSD were not always easy recognisable – this suggest that total duration spend in CSD would be a better measure but then you report that duration doesn’t show a difference across song categories. Does that mean, that if there were displays to other songs they must have longer? (as the yes/no occurrence was higher during father playbacks)? Should this noticable difference among unfamiliar/fathers’ songs not be discussed in more detail? Related to this point: the additional references you list here to compare duration/frequency of CSD with other studies should be discussed in more detail (now disc. only mentions these studies) and help readers to understand which measure is chosen most often in the Bengalese finch and why.*

> There have been only a few studies in Bengalese finches that measured CSDs (Clayton & Pröve, 1989; Okanoya, 2004; Dunning et al., 2014). Clayton & Pröve and Dunning et al. analyzed the frequency of CSDs, defined as the number of display bouts, while Okanoya reported whether or not birds performed the display. One study briefly described in the text that the typical duration of a CSD bout is 2 seconds (Dunning et al, 2014). In our study, because there were individual variations in CSD bout duration, and not all subjects displayed CSDs to unfamiliar songs, we primarily chose the occurrence of CSDs as a measure. We then added the duration analysis in the first revision. We modified the manuscript to specify the reasons for our chosen analyses in the Methods section (pp. 12-14, lines 211-218, 222-223, 224-231).

To clarify, we calculated the total CSD duration per trial as a measure of response intensity within a trial (one song presentation), and the value was averaged across all trials in which the bird showed CSDs to a given stimulus. Thus, the mean duration reported in the text and Fig 2 is not the cumulative duration throughout the test series (we assume this is what you meant in your comment). Considering that the mean duration of CSDs was longer for the father’s song than for unfamiliar song(s) in 6 out of 7 birds, we speculate that the response frequency and response intensity measured by duration are consistent with one another. In other words, even though the difference in CSD duration between stimuli did not reach statistical significance, there was still a trend in which CSD bouts were longer for the father’s song. We hope that the revised text (p. 13-14, lines 224-231; p. 17 lines 285-289 & 295; pp. 23-24, lines 398-400) now addresses your concerns.

*3) Glmm analyses: song presentation order within a test is now a fixed factor and the repeated testing days/events (test number) a random factor. This means that you are asking the model i) to check whether each position within the test order is different from any of the other (ignoring that there is an order/time effect from the 1^st^ to 5^th^ position) and ii) that your analyses do not address the question of whether order and stimulus type might show an interaction. Likewise, ‘test’ is now a random factor rather than a covariate meaning that also for this variable you again omit testing of whether order or and temporal effects occur within a test series. In short, neither within a test nor in the test series, is order or time controlled statistically. The current analyses – by treating test as a random variable – will not detect an order or time effect. The reason you give in the reply for not checking for an order/time effect is saying you could not predict the direction of the effect but this argument is irrelevant- you want to test whether repeated testing leads to an effect over time (should this be the case; data inspection can show you whether there was an in- or decrease).*

> Thank you for your comments. We noticed that there were still several points to be clarified in the manuscript regarding the details of the statistical analyses. Here, we give an explanation for each issue you raised:

**1. The presentation order (1-5) within a test is not controlled:** Since the presentation order was treated as a numerical variable, but not as a categorical factor, order and time information is retained. In other words, the GLMM has the potential to describe whether the birds’ responses could increase or decrease in earlier vs. later trials. We further clarified this type of variable (numerical) in the methods section (p. 15, lines 257-258).

**2. The model does not test the interaction of stimulus type and presentation order:** In the first round of the review, we explained why we decided not to include the interaction term. Please refer to our response on page 11 of the previous letter. In short, inspection of the data suggested an interaction such that the effect of presentation order was more significant in trials of unfamiliar songs. When we tried another model which included the interaction term, this resulted in a singular fit. However, even though we chose these model parameters due to a technical issue, the interaction is partly dealt with through another analysis of stimulus similarity (Fig. 3 & Table 2). Here, a GLMM was constructed to describe the factors that possibly predict the birds’ response to unfamiliar songs, and the presentation order was included as a fixed effect (again, the information of order was retained). The estimated parameters indicated a significant effect of presentation order on the occurrence of CSDs, which means that the birds showed more displays to unfamiliar stimuli presented earlier. Thus, our analyses as a whole, give substantial information about the possible interaction of stimulus type and presentation order, as far as our sample size allows.

**3. The test number should be treated as a fixed effect (but not as a random effect):** In the previous round of the review, we answered that it was difficult to simply assume an increase or decrease in response during the test series. Here we show the number of CSD occurrences of each bird throughout the experiment. There was no systematic change in the response frequency across individuals, suggesting that testing the effect of test number would not provide any additional information. In addition, from a technical/practical point of view, considering the overall low response rate and the individual variation in the number of tests required to finish the experiment, using a complex model with a relatively small number of subjects would likely result into an overfitting or a failure of parameter estimation. In sum, although the test number was not included as a fixed effect independent variable, the actual data indicates that doing so is not critical to the validity our statistical analysis.

In the graphs above, the total number of CSD occurrences in a test is plotted against the test number (from day1 to day5-9, depending on the individual). Each panel shows data from one female. We could not find a systematic increase or decrease in the frequency of displays throughout the test series.

*Additional specific comments by line number*

*20-21 add ‘if the father’s song is the only song they heard’ or mention that you want to know whether it is possible at all? As is you ask for a function of something that might be an artefact of the procedure, the question of function/consequence should not be about this specific song, but whether any song (incl father) heard during this time leads to a preference? (on first principles: if there is preference learning and females learn during a sensitive phase, then if there is only one thing to learn they are likely to learn (cf. ducklings imprinting on footballs) and if these learned preferences normally affect mating preferences this is likely also the case to involve the father’s song?) Here you ask if such preference lead to matings but this has already been shown in Bengalese finches – so you should be specific here saying it is to test whether this would also happen with the father’s song?*

> To ensure that our description of the research purpose is accurate, we changed the sentence as follows: ‘In the current study, we aimed to test whether the preference for the father’s song as reported in previous Bengalese finch studies, can be interpreted as a mating preference’ (p. 2, lines 20-24). Please also refer to our response to your major comment #1 for clarification of the research purpose. In addition, in Bengalese finches (as well as in zebra finches), the effect of early-life song experience on song preference at the species level has already been demonstrated by CSD assays (Clayton & Pröve, 1989), but no studies have shown that females prefer the actual (foster) father’s song to other conspecific songs. We realized that the contextualization was inadequate in the previous manuscript and revised the description in the abstract (p. 2, lines 20-24), and Introduction section (p. 4, lines 58-65 & p. 5, lines 74-77)

*27 this conclusion is too strong. Again, be clear about the reductionist environment: to be sure that this statement is correct you first would have to test if this effect also occurs if the father isn’t the only tutor and if females are raised in a more natural/more socially varied environment (as you suggest in the sentences after). Adjust the statement here so it is not general but that this preference is seen in the current circumstances and make clear that to test if a learned preference for the father’s song is relevant for mate choice it should also been seen if other songs were also heard, or should likewise be seen if the females had been exposed to another song (e.g. a foster father, cage mates during the sensitive phase etc..).*

> Thank you for your comment. We revised the description in the abstract, taking into consideration the specific rearing environment of the current study (p. 2-3, lines 30-34).

*37 odd sentence, perhaps rephrase? (Species recognition is not something uniquely special to female songbirds but occurs across species – it is the very essence of a mating signal?)*

> This sentence simply means that female birds recognize species by song. We do not argue that the conspecific-selective response is unique to females (or songbirds for that matter). To disambiguate that point, we rephrased the sentence to mention that courtship signals in general are designed to provide information on the signaler’s species (p. 3, lines 44-45).

*38 independently*

> Thank you for the correction. We revised this (p. 3, line 48).

*39 These references do not support the statement. The sentence makes a statement about songbirds in general and that some species develop experience independent preferences but the quotes are two experimental studies in the zebra finch (the two references thus refer to only one species and this is a species where preference is NOT experience-independent, but learned to a large degree).*

> We realized that the description and references here were not concordant and changed the sentence (p. 3, lines 46-47). For the latter indication, we discuss the selectivity for species-specific song features (but not about all the aspects of song preference), and the cited papers (Braaten & Reynolds, 1999; Lauay et al., 2004) both demonstrated the preference for zebra finch song vs. heterospecific song in song-naïve females. We modified the sentence so that the point is correctly conveyed.

*47 or another male’s/males’ song? (can be new/different adult male or from peer group)*

> We changed the description so that the father’s song is just one possible song that females may learn to prefer after early exposure (p. 4, line 57).

*48ff please check the literature again: there is plenty of evidence for several species (and in particular the zf which you discuss here) that these learned preferences affect mate choice/differential allocation etc.. to say here it has not been tested ignores a lot of previous work (zebra finch, cowbirds, white crowned sparrow, song sparrow, etc.. etc..)*

> Thank you. We believe our phrasing was not precise enough to sufficiently explain our logic. The issue we would like to address here is that when preference is measured by behaviors that are also expressed outside of a reproductive context, this does not necessarily mean that such preference is relevant to mating (e.g., it is possibly a more general preference for familiar stimuli). Thus, please understand that we do not argue that there is a lack of knowledge as to whether learned preferences in general affect mate choice. Instead, we argue that the choice of measurement affects what we can infer about preference. We hope that the revision here (p. 4, lines 58-65), together with related sentences in the abstract (p. 2, lines 20-22), better describe the position of this study compared to previous studies.

*50 again you are only citing two zebra finch studies while making general statements about song birds – what about e.g., Darwin finches as example for pref. for father’s song?*

> Thank you for your suggestion. We added a reference on sexual imprinting in Darwin finches (Grant & Grant, *PNAS*, 2010 on p. 4, line 61).

*56-58 you can’t address this here – drop?*

> As you say, in this study we cannot address the fitness benefit of preferring the father’s song. This sentence was written, not to deal with this question, but to justify the significance of carrying out this study. We rephrased this and the following sentences to correctly convey our intention in this context (p. 5, lines 68-72). (To provide details here, we thought that it seems maladaptive for females to actually learn to prefer their father’s song as a mating signal. Choosing a male who sings exactly the same song as the female’s father may lead to inbreeding, which can greatly affect the health of the offspring. Thus, even though previous studies have shown with some types of song stimuli that the song preferences measured by general behaviors such as phonotaxis or operant responses are relevant to mate choice, it is still unclear as to whether this also applies to the father’s song. We will not know the answer until preference is measured by behaviors that are expressed specifically in the context of reproduction (i.e., CSDs).)

*302 this is misleading – it should be preference for the song(s) they heard early in life – we do not know whether outside the limited exposure in the laboratory (only the father is available as tutor) which model (or several models) are influencing preferences*

> We completely agree with your comment. The sentence is now revised (p. 20, line 336).

*303 sweeping statement (and not correct) others have tested in both species whether song preferences translate to mate preferences – so please be specific! Perhaps what you want to say is that in BF that if such a preference for the father’s song exist that it will also lead to more CSD? It has definitely been shown for both species that song preferences (measured with a variety of methods) predict live male preferences/mating/pair formation.*

> We admit that the description needs to be more specific and changed the sentences (pp. 20-21, lines 336-346). Please also refer to our response to your comment on lines 56-58 for the reason we revised the sentences this way.

*316 Nicky Clayton’s work on cross fostering T.g.g and T.g.c. has shown that the females imprint on the father’s subspecies song and choose mates accordingly (the series of experiments is reviewed in (Clayton 1990).*

*Clayton NS 1990: Assortative mating in zebra finch subspecies, Taeniopygia guttata guttata and T. g. castanotis. Philosophical Transactions of the Royal Society of London Series B-Biological Sciences 330: 351-370. 10.1098/rstb.1990.0205*

> Thank you for the comment. Although sexual imprinting at the (sub)species level has already been shown in zebra finches by Clayton (1990), it is not clear if females acquire a sexual preference for the very song (among other conspecific songs) they heard. We should have been more specific about this and thus modified the sentence accordingly (pp. 21-22, lines 360-363).
