# Supplementary material for: Auditory and sexual preferences for a father’s song can co-emerge in female Bengalese finches
Source: PLoS One. 2022 Mar 10;17(3):e0254302. doi: 10.1371/journal.pone.0254302 (PMC8912213; doi:10.1371/journal.pone.0254302)
Supplement: Attachment — Submitted filename: Response to Reviewers(Fujii&Okanoya_PONE)round3.docx [file pone.0254302.s006.docx]

**General comments:**

Thank you for your suggestions to improve the manuscript. We hope that the revised title, abstract, and the main text now address your concerns. Please see below for our specific responses. Line numbers refer to the marked-up version of the revised manuscript.

**Journal requirements:**

*Please review your reference list to ensure that it is complete and correct. If you have cited papers that have been retracted, please include the rationale for doing so in the manuscript text, or remove these references and replace them with relevant current references. Any changes to the reference list should be mentioned in the rebuttal letter that accompanies your revised manuscript. If you need to cite a retracted article, indicate the article’s retracted status in the References list and also include a citation and full reference for the retraction notice.*

> We corrected the reference list based on the comment from the reviewer #2 and changed 2 references which give links to the figshare repository, since the article title has been modified (p. 29, refs 27 & 31).

**Additional Editor Comments:**

*TITLE: The reviewer reiterates a concern about the implications of the title; i.e., that some may misinterpret the scope of the study when reading the title. While I think the title previously proposed by the reviewer might be too limited in scope, I agree that even the revised title is too broad. In my opinion, including modifiers such as “can” and descriptors such as “experimental” would appease the reviewer and my concern about the scope of the title and would communicate the major thrust of paper. I propose some alternative titles that I believe satisfy everyone’s concerns and intentions:*

*o “Preferences for their father’s song can manifest themselves as sexual preferences in female Bengalese finches”*

*o “Auditory and sexual preferences for a father’s song can co-emerge in female Bengalese finches”*

*o “Experimental co-development (or co-emergence) of auditory and sexual preferences for a father’s song in female Bengalese finches”*

*ABSTRACT: Related to the above point, numerous readers might only read the abstract. Therefore, it is important to clearly emphasize the conditions of this experiment and the scope of interpretations in the Abstract. Although there is a conclusion about the limitations of this study at the end of the abstract, the design should be made explicit early in the abstract. Therefore, the sentence should be changed in the following way (my edits in CAPS): “For this purpose, the subjects were RAISED EXCLUSIVELY with their family (I.E., DID NOT HEAR THE SONGS OF OTHER MALES) until they became sexually mature and THEN TESTED AS ADULTS.” With regard to word limits, I am confident that other parts of the abstract can be streamlined (e.g., last two sentences) to accommodate the important clarifications about the experimental design in the sentence discussed above.*

*o NOTE: you need to specify the meaning of the acronym CSD (on line 25)*

> Thank you for the concrete suggestions. We modified the title and abstract so that it does not mislead the readers. We followed most of your suggestions to revise the abstract (p. 2, lines 22, 23, 27, & 29), but omitted the part “*(I.E., DID NOT HEAR THE SONGS OF OTHER MALES)”*, since the subject females could hear other males housed in separate cages placed in the same room (but certainly could not see nor interact with them).

*• Include the citations about zebra finch phonotaxis and preferences as stated by the reviewer.*

*• Be sure to add details about the upbringing when describing the results (e.g., when ‘…attracted to their father’s song ‘add ‘if it was the only song tutor’?)*

*• I agree with the reviewer that the figure on page 5 of the response to reviewers would be useful to include as a figure in the main text or as a supplementary figure.*

*• I strongly suggest making other the editorial suggestions made by the reviewer.*

> We changed the manuscript incorporating all these suggestions from the reviewer #2. Please see the following responses for details.

**Reviewer #2:**

*The authors wrote a detailed reply and made an effort to accommodate the comments.*

*Please find below a few last suggestions you might find hopefully useful.*

*Title: I understand your rationale for your tests and the abstract and introduction make this much clearer now, but the new title could still prime readers in the wrong way. The new title reads as if you found and describe a general property of BF mating preferences (which may or may not be the case as you had a very limited social setting in the experiment) – while a title that would acknowledge the limited song exposure they had (e.g., “female BF finches reared with father as sole song tutor show…” or similar would be a better summary of the study).*

> We understood your concern about the generality of the current results. We changed the title to “Auditory and sexual preferences for a father’s song can co-emerge in female Bengalese finches” as suggested by the Academic Editor. The short title is also revised (new title: “Auditory and sexual preferences for a father’s song in female Bengalese finches”).

*67/8 in zebra finches several studies have shown that song preferences in phonotaxis or operant tests translate to preferences in real males and that the preferences in such association tests predict pair formation (Clayton, Witte work for example).*

> We now added the reference ([19] Witte, *Ethol Ecol & Evol*, 2006) in the Introduction (p. 4, line 64) and Discussion (p. 20, line 334).

*129 here or discussion: hormone implants increase motivation but might also change preferences see for z.f.*

> We additionally discussed about the possible effect of hormone implants, referring to Vyas et al., *Horm Behav*, 2009 (p. 21, lines 347-350), and slightly modified another sentence accordingly (p. 20, lines 339-340).

*318 ‘recognize’ doesn’t seem the right word here – ‘perceive’?*

> We replaced the term ‘recognize’ with ‘perceive’ as you suggested and modified the sentence according to your comment to line 347 and the editor’s advice (please see below; p. 18, lines 310-311).

*337-338 change link bewteen sentencese: the sentence in 338 starts with ‘these’ which refers to (several types of) studies in the sentence before but sentence starting in 338 is only about father’s song, so ‘these’ is not correct here.*

*340 ‘Other studies’ perhaps better ‘In several other species..’*

*342 this is one zf study and one swamp sparrow study but from context it is not clear whether you are talking about BF or other species here*

> To collectively address these problems, we revised the sentences (p. 20, lines 330 & 332). As the original description “their father’s song” (the boldfaced part in the sentence “These studies of female preference specifically for **their father’s song** measured…”) was wrong, we rather changed this part instead of the link between sentences. We also added the reference here ([19] Witte, *Ethol Ecol & Evol*, 2006) that you indicated in the comment to the Introduction section (line 334).

*347 ‘…attracted to their father’s song ‘add ‘if it was the only song tutor’?*

> We inserted the rearing condition here (p. 20, line 339) and made a similar change in a following sentence (line 341).

*356 is it the lab setting or the single tutor? (Situation in the lab could be similar to the wild if there were many tutors? if there is a study in the lab that can emulate social situation in the wild sufficiently you might have the same song learning although it is in the lab)*

> We acknowledge that the lab/wild distinction might not be appropriate here, and rephrased it as follows: ‘…might be captured in this unique experimental setting, but not necessarily in other social environment as discussed later in this section.’ (p. 21, lines 351-353)

*361 especially in the zf there are examples not only for species preferences but also specific songs…*

> We inserted a phrase here to clarify what we meant by the previous sentence (the sentence begins with ‘Although females preferred songs of the same species as their foster father, …’) (p. 21, lines 359-360)

*The figure on page 5 in the reply is very interesting, as a reader I would appreciate seeing it either in the manuscript or in an appendix*

> We included this figure as a new supplementary file (S3 File; p. 33, lines 561-562) and referred to it in the Method section (p. 11, lines 191-192).

*Ref list*

*- species names in italics*

*- caps in Bengalese not used consistently*

> We reviewed and corrected the spelling on the species names (refs 3, 17, 36, 47, 51, & 55).
